# *Crocus sativus* improves cognitive functions and reduces anxiety-like behavior in aged rats exposed to chronic cold temperature-induced stress

**DOI:** 10.3389/fnmol.2026.1872086

**Published:** 2026-07-22

**Authors:** Vijayan Priya, Faraz Ahmad

**Affiliations:** Department of Integrative Biology, School of Biosciences and Technology, Vellore Institute of Technology, Vellore, India

**Keywords:** brain aging, low temperature stress, memory impairment, neurobehavior, phytotherapeutic

## Abstract

**Introduction:**

Chronic cold stress acts as an influential environmental stressor that disrupts neuro-behavioral functions, which include anxiety responses, cognitive performance, mood regulation, and stress susceptibility. In spite of the growing recognition of cold-induced neuropathology, effective therapeutic strategies remain limited. *Crocus sativus* (saffron) extract, and its principle bioactive constituents (crocin, crocetin, safranal) have emerged as effective neuroprotectants. They elicit potent antioxidant and anti-inflammatory properties and have beneficial effects on cognitive functions, memory, and anxiety-related behaviors. However, their potency in cold-induced neuropathology remains obscure.

**Methods:**

Structured phytochemical characterization of saffron extract was performed using UV-visible spectroscopy, Fourier transform infrared spectroscopy, gas chromatography-mass spectrometry, and high-performance liquid chromatography. The antioxidant profile of the extract using 2,2-diphenyl-1-picrylhydrazyl radical scavenging activity was analyzed, along with the assessment of total phenolic content, total flavonoid content and moisture content. Chronic cold exposure was induced at 4°C for 30 min/day over a period of 4 weeks. Neurobehavioral parameters of general exploration, anxiety, and memory were evaluated using established behavioral assessment paradigms of open field, elevated plus maze, Y-maze, and novel object recognition, respectively.

**Results:**

Phytochemical characterization of saffron extract confirmed the presence of major bioactive constituents, including crocin and safranal. Additionally, a high phenolic and flavonoid content was supported by a robust antioxidant activity *in vitro*. Behavioral analysis revealed that cold stress significantly impaired memory and increased anxiety-like behavior, whereas oral supplementation of saffron extract reduced anxiety, improved spontaneous alternation and enhanced object recognition memory.

**Conclusion:**

Saffron extract enhances neurobehavioral functions, significantly ameliorating cold stress-induced cognitive deficits and anxiety-like behavior. While these findings support its neuroprotective potential in stress-related neuropsychiatric disorders, the underlying mechanisms require further investigation.

## 1 Introduction

Over the past decades, stress has been recognized as a dynamic, adaptive neurobiological process involving complex brain-body interactions that regulate environmental responses. Under chronic conditions, stress leads to neuroplastic alterations, which further increase vulnerability to neurological conditions such as depression (Albernaz-Mariano et al., 2025; Lei et al., 2025) and cognitive impairment (McEwen and Akil, 2020; Yaribeygi et al., 2017). Stress can impact nearly every system in the body, with adverse effects on both behavior and physiological functioning. Indeed, studies have consistently demonstrated that stress disrupts cognitive processing, recognition memory (Santori et al., 2020) and alters exploratory patterns (Hoffman et al., 2011). Research studies have demonstrated the stress-induced physiological and behavioral alterations in both humans (McEwen, 2006; van der Kolk, 1994) and animals (Bowman et al., 2003). Further, stress can induce persistent, age- and exposure-dependent structural and functional brain alterations, accelerating cognitive decline and neurodegeneration (Ben-Azu et al., 2024; Kline and Mega, 2020).

Chronic stress, particularly cold temperature stress, is an exacerbating environmental factor that has been shown to induce significant behavioral alterations (Muller et al., 2012; Watkins et al., 2014), including reduced locomotor activity, impaired exploratory behavior, and increased anxiety-like responses in animal models (Değirmenci et al., 2024; Khattak et al., 2023; Suresh et al., 2013; Priya and Ahmad, 2026). Several human studies have also demonstrated that chronic or acute cold exposure impairs cognitive performance and increases neurophysiological strain, particularly affecting attention, processing speed, executive function, decision-making, and memory (Falla et al., 2026; Martínez Lozada and Leon-Rojas, 2025). Chronic cold stress disrupts physiological homeostasis and thermoregulation, characterized by decreased vasoconstriction, lower metabolic heat production, and reduced thermal sensitivity, leading to greater core temperature decline and increased systemic vulnerability predominantly in aging populations (Smolander, 2002). Interestingly, the exposure to cold stress may affect the behavior in an intensity-dependent manner. For instance, it has been observed that mild intensities of cold exposure enhance exploration and reduce anxiety; however, prolonged exposure may lead to reduced exploration, impaired movement, and elevated anxiety-like responses (Hu et al., 2022).

The adverse behavioral impairments associated with cold stress necessitate the need to explore natural, phytotherapeutic interventions (Mishra and Jain, 2025). *Crocus sativus*, commonly known as saffron, contains a rich profile of antioxidant and anti-inflammatory bioactive carotenoids and terpenoids, including crocin and safranal (Jahromi et al., 2023). These bioactive compounds are recognized for their anxiolytic and neuroprotective properties (Asgary, 2021; José Bagur et al., 2017; Ziani et al., 2025), collectively supporting saffron’s potential benefits for brain health (Cerdá-Bernad et al., 2022). Across multiple preclinical models, crocin and saffron extract (SE) have been shown to reduce depressive and anxiety-like behaviors, as evidenced by improved performance in behavioral tests assessing mobility, anhedonia, exploration, and emotional responses (Tao et al., 2023; Abbaszade-Cheragheali et al., 2022; Siddiqui et al., 2022). Additionally, SE reverses stress-induced dysfunctions in learning, memory, and cognition (Ghadrdoost et al., 2011). Similarly, using an aged rodent model, reported that saffron supplementation improved cognitive performance and anxiety-like behaviors, highlighting its potential as a multi-target intervention against age-associated neurobehavioral decline (Navarro et al., 2026). Clinical findings indicate that SE is at least as effective as standard drugs for depression and anxiety, and improves cognitive outcomes with good tolerability (Chauhan et al., 2024; Yang et al., 2018). Importantly, SE has been envisioned as a potential therapeutic against brain aging-related dysfunctions, including neurodegenerative and cardiovascular diseases (El Midaoui et al., 2022; Li et al., 2025).

Regardless of the extensive findings of the beneficial effects of SE under psychological stress, the present study introduces a distinct perspective by investigating its ability to counteract cold stress-induced neurobehavioral dysfunction specifically triggered by an environmental (cold stress)-aging axis. Further, the study bridges a critical gap between environmental neurobiology, gerontology, and phytotherapy research, highlighting the possibility of SE as a potential therapeutic agent either alone or in complementation with other strategies.

## 2 Materials and methods

### 2.1 Chemicals

Ethanolic extract of stigma of *Crocus sativus* L. used in this study was from Kancor Ingredients Pvt. Ltd., India, a certified manufacturer of plant-derived ingredients. All the chemicals utilized for the study were of analytical grade. 2,2-diphenyl-1-picrylhydrazyl (DPPH) was purchased from Sisco Research Laboratories (SRL) Pvt. Ltd., India. All other reagents were of research grade and were obtained from local suppliers.

### 2.2 Animals

Adult male Wistar rats (18–20 months old), 280–320 g in weight, were selected for conducting the study. Animals were maintained on a 12/12 h light/dark cycle regimen at a temperature of 22°C in the animal housing facility of Vellore Institute of Technology (VIT), Vellore, India. Food and water were available *ad libitum*. Extra care was taken to minimize animal suffering and to limit the number of animals used for experiments. For the study, all treatments and behavioral testing were performed in the mornings (between 08:00 and 12:00) to minimize the influence of circadian rhythms. All experiments were approved by the Institutional Animal Ethics Committee, Vellore Institute of Technology (VIT), India (IAEC; approval no. VIT/IAEC/18/Dec 24/09; dated 18 December, 2024).

### 2.3 Cold stress induction and treatment protocol

Aged Wistar rats were randomly assigned to four experimental groups: control, cold stress (CS), cold stress + saffron extract (CS+SE), and saffron-only (SE) groups. The chronic intermittent cold stress paradigm used in this study was based upon previous studies (El Marzouki et al., 2021; Jayakumar et al., 2017), and involved exposure of animals to a cold environment maintained at 4°C for 30 min/day for a period of 4 weeks. Animals were individually exposed to the cold environment in cages without bedding to prevent thermoregulation through huddling and insulation-mediated heat retention. After ending each of the cold stress session and upon their return to room temperature, animals in the CS+SE group were administered SE at a dose of 150 mg/kg body weight via oral gavage (Hosseinzadeh et al., 2012; Kim et al., 2023). The SE group were administered the same dose without stress exposure for the period of 4 weeks. Body weights were recorded weekly, and dosing volumes were adjusted accordingly. Followed by the 4-week treatment period, animals were allowed a 5-day washout period, and then proceeded for behavior analysis.

### 2.4 Phytochemical analysis

#### 2.4.1 UV-visible spectral analysis

The UV-visible spectroscopic analysis was performed to confirm the presence of crocin (Mir et al., 2022), a characteristic carotenoid compound in SE. The wavelength range obtained from the absorption spectra was 300–500 nm with a scan interval of 0.2 nm.

#### 2.4.2 Fourier transform infrared (FTIR)

FTIR spectroscopy was carried out on an FTIR spectrometer (Agilent Cary 630) to determine the relevant functional groups present in SE. Spectra were recorded between 4,000 and 400 cm^–1^ with a resolution of 2 cm^–1^. Spectra obtained were analyzed to identify characteristic absorption bands corresponding to bioactive functional groups.

#### 2.4.3 High-performance liquid chromatography (HPLC)

HPLC was performed to determine the presence of one of the other major constituents, safranal, in SE. The absorbance was recorded at 330 nm, which corresponds to the maximum absorbance of safranal. The presence of safranal was analyzed using the retention time (RT) of peaks obtained from the extract against the pure standard safranal.

#### 2.4.4 Gas chromatography-mass spectrometry (GC-MS)

GC-MS was performed to reveal the chemical composition of SE. The sample was injected in split mode. Helium was used as the carrier gas at a constant flow rate. The recorded mass spectra were analyzed using the NIST Mass Spectral Library for the identification of candidate compounds. Based on the peak area normalization, the relative percentage of each compound was calculated.

#### 2.4.5 Moisture content

The moisture content of the SE was determined by following Karl Fischer (KF) titration method (Scholz, 1984), a specific technique for quantitative water estimation. 10 mg of SE was introduced into the titration vessel, and analysis was performed using an automated KF titrator based on the volumetric titration principle. The endpoint was detected by the instrument, and the moisture content was calculated based on the volume of KF reagent consumed. Results were expressed as percentage (% w/w) moisture content of the sample.

### 2.5 Antioxidant assay

DPPH assay was performed to examine the *in vitro* antioxidant activity of SE. A previously published protocol (Frusciante et al., 2024) was used with slight modifications. Briefly, 1 mL of SE at varying concentrations (10–100 μg/mL) was mixed with freshly prepared 1 mL of ethanolic solution of 0.1 mM DPPH. The mixture was incubated for 30 min at room temperature in the dark to reduce photo-degradation of the radical. After incubation, the decrease in absorbance was measured at 517 nm using an UV-visible spectrophotometer (Shimadzu UV-1280). A negative control sample was prepared by mixing ethanol with DPPH solution. Experiments were carried out in triplicates. The radical scavenging activity was calculated as percentage inhibition of DPPH radicals, using the standard formula:


$$\%Inhibition=\frac{\left(A_{c\,o\,n\,t\,r\,o\,l}-A_{s\,a\,m\,p\,l\,e}\right)}{A_{c\,o\,n\,t\,r\,o\,l}}\times 100$$

### 2.6 Total phenolic content (TPC)

The TPC of SE was determined by Folin-Ciocalteu colorimetric method (Baliyan et al., 2022) with slight modifications. Briefly, 0.5 mL of 1 mg/mL SE was mixed with 2.5 mL of Folin-Ciocalteu reagent (diluted 1:10, v/v), and incubated for 5 min. After incubation, 2 mL of 7.5% (w/v) sodium carbonate solution was added to the reaction mixture. The mixture was then incubated for 30 min in the dark at room temperature. The absorbance was recorded at 765 nm using a UV spectrophotometer. A standard calibration curve of gallic acid over a concentration range of 20–100 μg/mL was used as the reference for calculating the TPC value of SE. The results were expressed as mg gallic acid equivalents (GAE) per gram of extract.

### 2.7 Total flavonoid content (TFC)

The TFC of SE was determined by following aluminum chloride colorimetric method (Khodaie et al., 2012) with slight modifications. Briefly, 0.5 mL of 1 mg/mL SE was mixed with 2 mL of distilled water, followed by the addition of 0.15 mL of 5% (w/v) sodium nitrite. After 5 min of incubation, 0.15 mL of 10% (w/v) aluminum chloride was added, followed by an incubation period of 6 min at room temperature in the dark. Subsequently, 1 mL of 1 M sodium hydroxide was added, and the final volume was adjusted to 5 mL with distilled water. The reaction mixture was mixed thoroughly, and the absorbance was measured at 510 nm. A standard calibration curve of quercetin over a concentration range of 20–100 μg/mL was used for calculating TFC of SE. The result was expressed as mg quercetin equivalents (QE) per gram of extract.

### 2.8 Neuro-behavioral assessment

#### 2.8.1 Body weight

To study the impact of chronic cold stress and treatment on body physiological status, body weight of animals of all experimental groups was recorded and monitored periodically. Animals were weighed every week starting at baseline (day 0) and subsequently on days 7, 14, 21, and 28 throughout the experimental period using a calibrated digital balance. The recorded data were used to calculate the % decline in weight during and after the stress exposure and treatment paradigms. After a washout period of 5 days, neurobehavioral assessment for all rats were performed as described below.

#### 2.8.2 Elevated plus maze (EPM)

The elevated plus maze (EPM) is a widely used behavioral test that evaluates anxiety-like behavior in rodents based on their natural avoidance of open, exposed spaces. The apparatus, as originally described by Pellow et al. (1985), consists of a plus-shaped maze made of black Plexiglas. It includes two closed (CA) and open (OA) arms (41 × 15 × 29.5 cm) arranged opposite to each other, with surrounding walls, with a slightly raised edge (1 cm) to prevent the animals from falling. The test was initiated by placing individual rats in the central part of apparatus with the head facing one of the closed arms. The rats were allowed to explore the maze freely for a duration of 5 min. The number of entries into open and closed arms, as well as the time spent in these arms, were measured to evaluate anxiety-related behavior in rats.

For EPM and all subsequent behavioral tests, recording of animal movements was performed using a camera positioned above the apparatus. Movements were tracked using Anymaze software (Stoelting, India). After each trial, to prevent the influence of olfactory cues, the mazes were thoroughly cleaned with 70% ethanol. Animals exhibiting minimal or no activity during the tests were excluded as outliers.

#### 2.8.3 Open field (OF)

The OF test assesses the general locomotor and exploratory activity of rodents, in addition to anxiety-like pattern in rodents (Campos et al., 2013). The OF apparatus consisted of a square arena (90 × 90 × 58 cm), with the floor divided into 16 equal squares (4 × 4 grid) to facilitate behavioral scoring. During the start of the experiment, each rat was placed in one corner of the arena with its head facing the corner (Jänicke and Coper, 1996), and allowed to freely explore for a duration of 5 min. The primary parameters analyzed included total pathlength (locomotor activity), pathlength and time percentages in the central (CZ), middle (MZ), and outer (OZ) zones.

#### 2.8.4 Y-maze

The Y-maze test is performed to check the spontaneous alternation, which assesses spatial working memory in rodents. The apparatus is in the shape of “Y,” consisting of three identical 49 × 8 × 40 cm arms (arbitrarily designated as A, B, C), separated from each other by 120° angles. Each animal was placed on the edge of the starting arm (A) and was allowed to freely explore the arena for 5 min. Rodents with intact spatial working memory recall recently visited arms and preferentially explore less recently visited ones, thereby exhibiting a natural tendency toward spontaneous alternation. In contrast, rodents with impaired working memory fail to retain information about prior arm entries, resulting in reduced alternation behavior and increased repetition (El Marzouki et al., 2021). A correct alternation behavior was operationally defined as successive entries into each of three arms as overlapping triplet sets (i.e., ABC, BCA, CAB, etc.) (Ahmad et al., 2021). The total number of entries into the arms of the Y maze and the number of triads were recorded to calculate the percentage of spontaneous alternation, which is used as a measure of spatial working memory performance.

#### 2.8.5 Novel object recognition (NOR)

The NOR test is a well-established behavioral test for evaluating recognition memory in rodents. The test was conducted in the OF arena with the animals habituated to the familiar objects (FOs). For these trial phases, rats were placed in the OF arena containing four objects positioned in each of the corners (Hale and Good, 2005). Rats were allowed to explore the objects (and the arena) freely. Object exploration was defined as a direct physical contact with it or directing the nose toward the object at a close distance ( ≤ 2 cm). After three trial sessions, the test phase was performed by replacing one of the familiar objects with a novel object (NO). Object recognition memory was evaluated by calculating the total object exploration time and percentage of time spent exploring FOs and NO.

### 2.9 Statistical analysis

All statistical data were expressed as mean ± standard error of the mean (SEM). Statistical comparisons were performed using one-way analysis of variance (ANOVA) followed by Bonferroni’s *post-hoc* test. For ascertaining the influence of two factors on a variable of interest (for e.g., animal groups and time on weight changes; and object type and animal groups on object exploring time in NOR tests), a mixed effects model with Geisser-Greenhouse correction, followed by Tukey’s *post-hoc* test was used. Of note, animals exhibiting immobility during specific behavioral tests were excluded to preserve the validity of the behavioral data. For all comparisons, F-ratios were calculated and tested for statistical significance at an alpha level of *p* < 0.05.

## 3 Results

### 3.1 Phytochemical profiling of *Crocus sativus* extract

**FIGURE 1:**
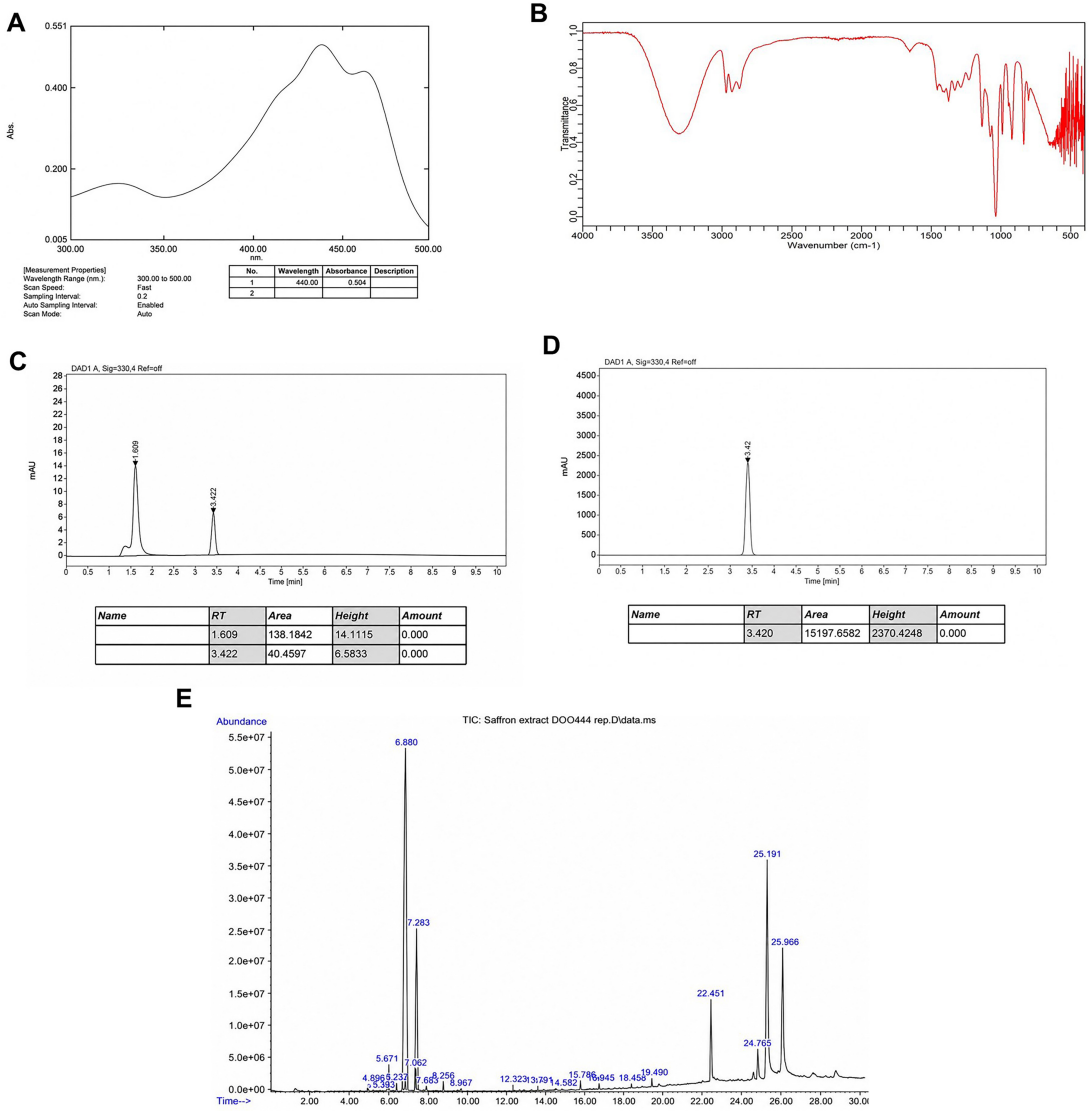
Phytochemical characterization of SE extract. **(A)** UV-Visible spectrum showing the characteristic absorption peak of crocin at 440 nm; **(B)** FTIR Spectrum; **(C)** HPLC chromatogram of Crocus sativus extract showing a corresponding peak confirming the presence of safranal; **(D)** HPLC chromatogram of safranal standard indicating retention time (RT); **(E)** GC-MS chromatogram depicting the profile of volatile constituents identified.

The major constituents of SE were evaluated through phytochemical characterization using UV-Visible spectroscopy, HPLC, and GC-MS analyses. The UV-visible spectrum of the extract showed a prominent absorption peak at 440 nm with an absorbance value of 0.504 (Figure 1A). Based on the previous study reports (Lage and Cantrell, 2009; Yamaguchi, 2012), this peak potentially corresponds to crocin, a major carotenoid pigment responsible for the color and bioactivity of SE.

The FTIR spectrum (Figure 1B) of the SE exhibited a broad absorption band around 3,300–3,400 cm^–1^ corresponding to O-H stretching vibrations (Coates, 2006; Stuart, 2004), suggesting the presence of hydroxyl groups in phenolic and glycosidic compounds (Alnaddaf et al., 2023; Naim et al., 2022b). The absorption peaks observed at 2,920 cm^–1^ were associated with aliphatic C-H stretching, while the band around 1,650–1,700 cm^–1^ corresponds to C = O stretching, indicating the presence of aldehydic constituents such as picrocrocin and safranal. Additionally, absorption bands in the regions of 1,600–1,500 cm^–1^ and 1,200–1,000 cm^–1^ potentially correspond to aromatic C = C and C-O stretching vibrations, further supporting the presence of phenolic structures and glycosidic compounds such as crocin (Foschi et al., 2022; Naim et al., 2022a). Collectively, consistent with the phytochemical composition of SE, the FTIR profile confirmed the presence of key functional groups.

To identify and quantify the presence of safranal, HPLC analysis of the extract was performed. The presence of the compound was confirmed by comparing the chromatographic peak of the extract against a standard safranal reference. The standard safranal exhibited a sharp and well-resolved peak at a retention time (RT) of 3.420 min (Figure 1C), representing its characteristic elution under the standard chromatographic conditions. SE showed a corresponding peak at RT of 3.422 min (Figure 1D), comparable to the standard, indicating the presence of safranal. Quantitative analysis using an external standard calibration curve revealed that safranal constituted 0.17% (w/w) of the extract, indicating its appreciable presence as a major volatile component. In addition, the early eluting peak observed at 1.609 min may be attributed to crocin/crocetin esters, predominant and highly polar constituents of SE, which elute earlier than less polar compounds such as safranal (Alam et al., 2016; Valle García-Rodríguez et al., 2014). Overall, the HPLC results confirm both the identity and quantifiable presence of safranal, supporting the phytochemical integrity and potential biological activity of SE.

GC-MS analysis showed the complex profiling of SE, revealing multiple volatile and bioactive constituents present in the extract (Figure 1E and Table 1). A total of 21 compounds were identified based on their retention time and mass spectra using the NIST mass spectral library. Among the identified compounds, safranal was detected at a retention time of 7.283 min, with a relative abundance of 3.25%, confirming its presence as a major volatile component of SE. Other notable compounds included 2,6,6-trimethylcyclohexa-1,4-dienecarbaldehyde, furfural, 2,5-dimethyl-3(2H)-furanone, and cyclopentenone derivatives, which are known contributors to aroma and biological activity of SE (Rezaee and Hosseinzadeh, 2013; Shafat et al., 2021). Additionally, minor constituents such as acetic acid, formic acid, caprylic acid, and hydroxymethylfurfural were detected in lower abundances. The presence of these compounds indicates a diverse phytochemical composition, including aldehydes, ketones, organic acids, and furan derivatives.

**TABLE 1:** GC-MS profiling of *Crocus sativus* extract.

Peak no.	Retention time (min)	Compound identified	Relative abundance (MS area %)
1	4.836	Acetone alcohol	0.09
2	5.671	Acetic acid	0.23
3	5.814	Furfural	0.04
4	6.031	Formic acid	0.10
5	6.076	2,6,6-Trimethylcyclohexa-1,4-dienecarbaldehyde	0.06
6	6.155	2,4-Dihydroxy-2,5-dimethyl-3(2H)-furan-3-one	0.06
8	7.062	β-Cyclocitral	0.31
9	7.283	Safranal	3.25
10	8.312	2,4-Dimethylbenzaldehyde	0.07
11	8.525	5(2H)-Furanone	0.15
12	8.578	Cyclopenten-1-one, 2-hydroxy-	0.03
13	9.108	2-Pentenoic acid	0.03
14	9.447	Acetal PG/Safranal (1)	0.12
15	12.383	2-Hydroxy-3,5,5-trimethylcyclohex-2-ene-1,4-dione	0.06
16	12.973	Caprylic acid	0.06
17	14.582	2-Hydroxy-gamma-butyrolactone	0.03
18	15.778	Pyranone	0.13
19	16.204	4-Hydroxy-3,5,5-trimethylcyclohex-2-enone	0.11
20	16.945	4-Hydroxy-2,6,6-trimethylcyclohex-1-enecarbaldehyde	0.12
21	18.456	5-Hydroxymethylfurfural	0.09

### 3.2 SE elicits potent DPPH-reducing antioxidant activity

**FIGURE 2:**
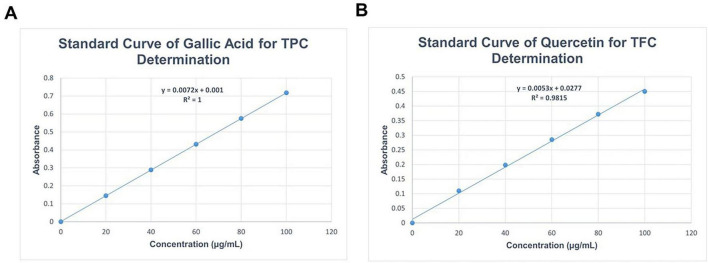
Standard calibration curves of TPC and TFC. **(A)** Standard calibration curve of gallic acid used for the determination of total phenolic content (TPC) of *Crocus sativus* extract. Data represent mean values of triplicate measurements. **(B)** Standard calibration curve of quercetin used for the determination of total flavonoid content (TFC) of *Crocus sativus* extract.

The quantitative phytochemical composition and antioxidant potential of SE were evaluated through estimation of TFC, TPC, DPPH and moisture content. The TPC of the extract was determined using the Folin-Ciocalteu method. As depicted in Figure 2A, the gallic acid standard curve demonstrated excellent linearity over the tested concentration range (20–100 μg/mL). Based upon the absorbance of SE at 765 nm, its TPC was calculated to be 75.13 ± 0.006 mg GAE/g. The TFC of SE was quantified using the aluminum chloride method at 415 nm. Quercetin was used as the standard reference and the calibration curve showed good linearity within the concentration range of 20–100 μg/mL (Figure 2B). Based on the calibration curve, the TFC of SE was calculated to be 41.94 ± 1.13 mg QE/g. The obtained results indicate the presence of appreciable levels of phenols and flavonoids in SE.

**FIGURE 3:**
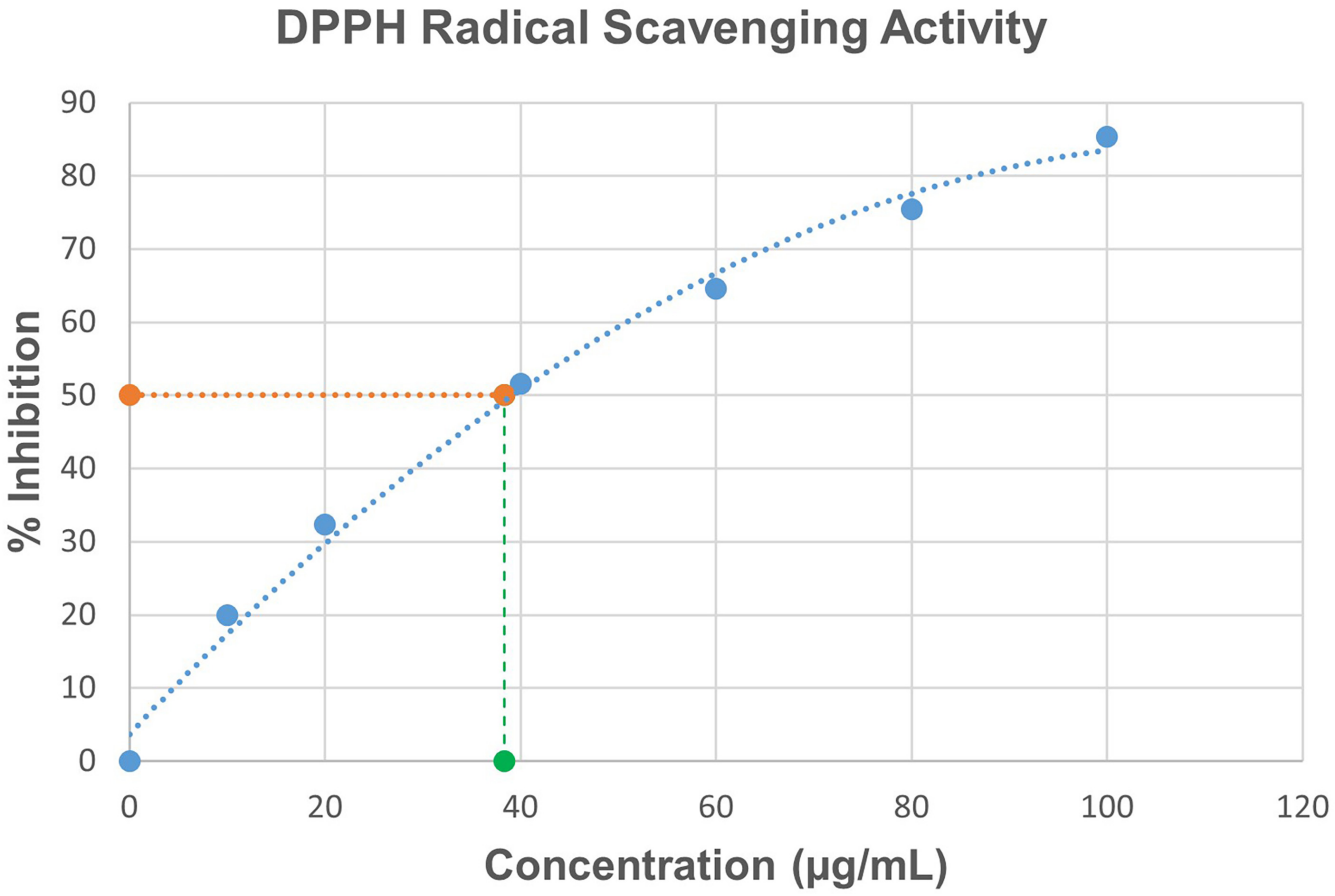
DPPH radical scavenging activity of SE extract at different concentrations. The IC_50_ value was calculated from the dose-response curve.

Antioxidant activity analyzed using the DPPH assay showed that SE exhibited a concentration-dependent increase in radical scavenging activity (Figure 3). The percentage inhibition progressively increased from 20% at 10 μg/mL to 85.38% at 100 μg/mL, indicating strong antioxidant potential. The extract achieved approximately 50% inhibition between 20 and 40 μg/mL, and the IC_50_ was determined by interpolation method to be 38.4 μg/mL. The observed antioxidant activity is consistent with the high TPC and TFC of SE. Finally, the moisture content of SE estimated using KF method was found to be 1.2% (w/w). The low moisture level indicates good stability and quality of the extract, as reduced water content minimize the risk of microbial growth and degradation of bioactive constituents and prolongs its shelf life.

### 3.3 Neuro-behavioral outcomes

#### 3.3.1 SE supplementation rescues cold stress-induced weight loss

**FIGURE 4:**
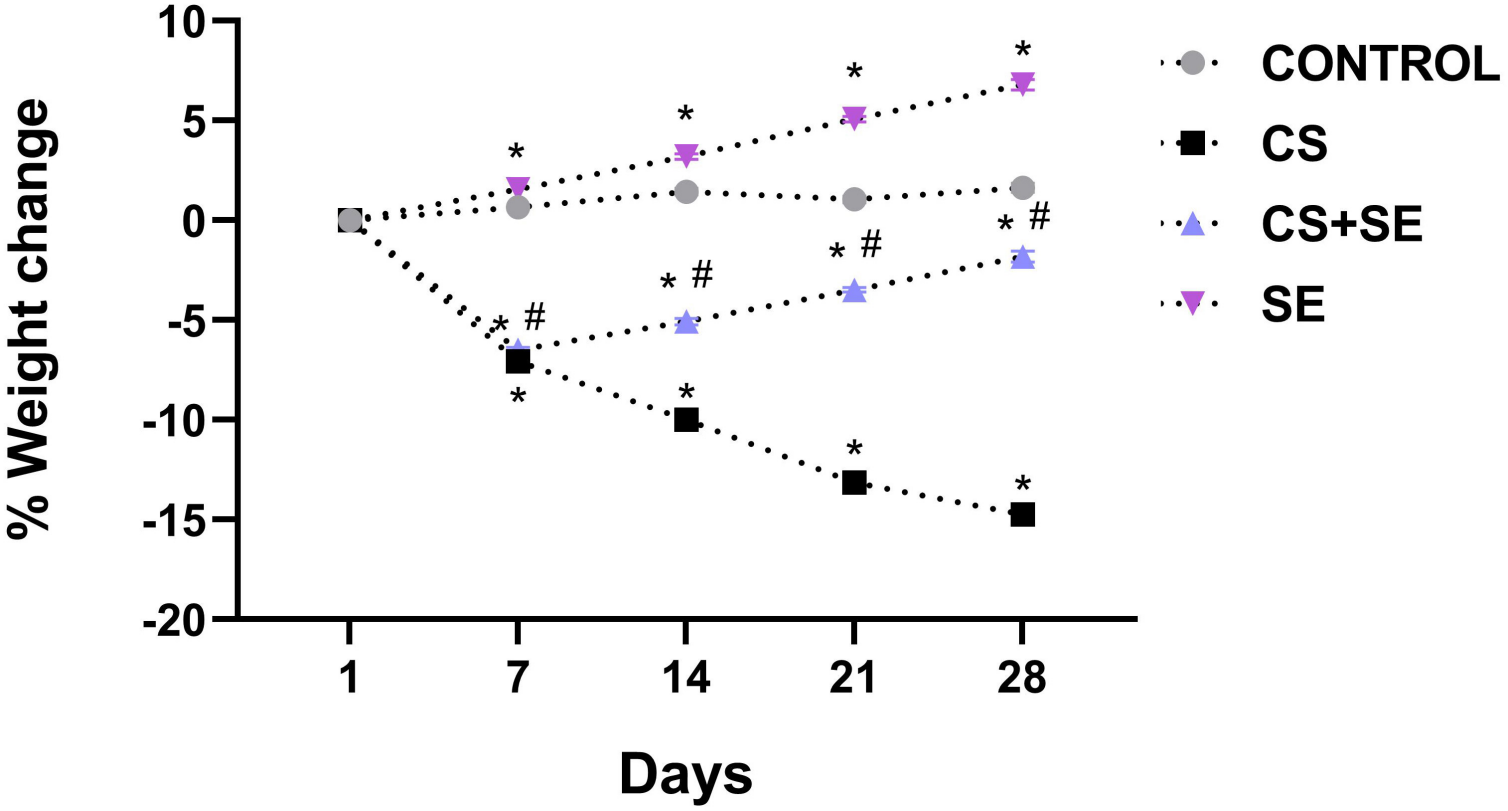
Body weight changes (%) in experimental groups: Effect of changes in control, cold stress (CS), stress + saffron (CS+SE), and saffron only (SE) groups over 28 days. Data are expressed as mean ± SEM. Statistical analysis was performed using a mixed-effects model (REML), followed by Tukey’s multiple comparisons test. Significant effects of group, time, and group × time interaction were observed (*p* < 0.0001). *p < 0.05 vs. control; #*p* < 0.05 vs. cold stress (CS) group.

Physiological effects of chronic cold stress and treatment on body weight were assessed over a period of 28 days (Figure 4). A significant effect of animal group [*F*(4,140) = 1,116, *p* < 0.0001], time [*F* (2.249, 105.0) = 33,362, *p* < 0.0001], and group × time interaction [*F*(12,140) = 3,447, *p* < 0.0001] on body weight was observed. CS group exhibited a progressive and marked decline in body weight throughout the experimental period, with percentage changes of −7.05% (day 7), −9.99% (day 14), −13.13% (day 21), and −14.73% (day 28). Tukey’s *post hoc* analysis revealed that the reduction in body weight was significant at all-time points compared to baseline, indicating a sustained negative impact of chronic cold exposure on physiological status. In contrast, the treatment (CS+SE) group showed a significant reduction in weight loss, showing a gradual recovery pattern over time. Although an initial reduction was observed (−6.49% on day 7), the decline was progressively reduced to −1.80% by day 28. Tukey’s *post hoc* analysis revealed significant differences across CS and CS+SE groups across all time points, reflecting a time-dependent recovery and suggesting a protective effect of saffron against stress-induced metabolic changes. The control group maintained a relatively stable body weight, with only minor fluctuations ranging from 0.66 to 1.64% across the study period. Notably, the SE group showed progressive gains of 1.56% (day 7), 3.20% (day 14), 5.08% (day 21), and 6.80% (day 28), demonstrating a consistent pattern of weight gain. The increase in body weight observed in the saffron-treated group is likely attributed to the natural macronutrient composition of crude SE, including carbohydrates, sugars, proteins, and lipids, as well as its reported effects on digestive efficiency and nutrient utilization (Khorasany and Hosseinzadeh, 2016; Melnyk et al., 2010; Mirhadi et al., 2020; Ríos et al., 1996). This might suggest that SE administration alone enhances normal growth and metabolic function.

#### 3.3.2 SE treatment has anxiolytic effects

**FIGURE 5:**
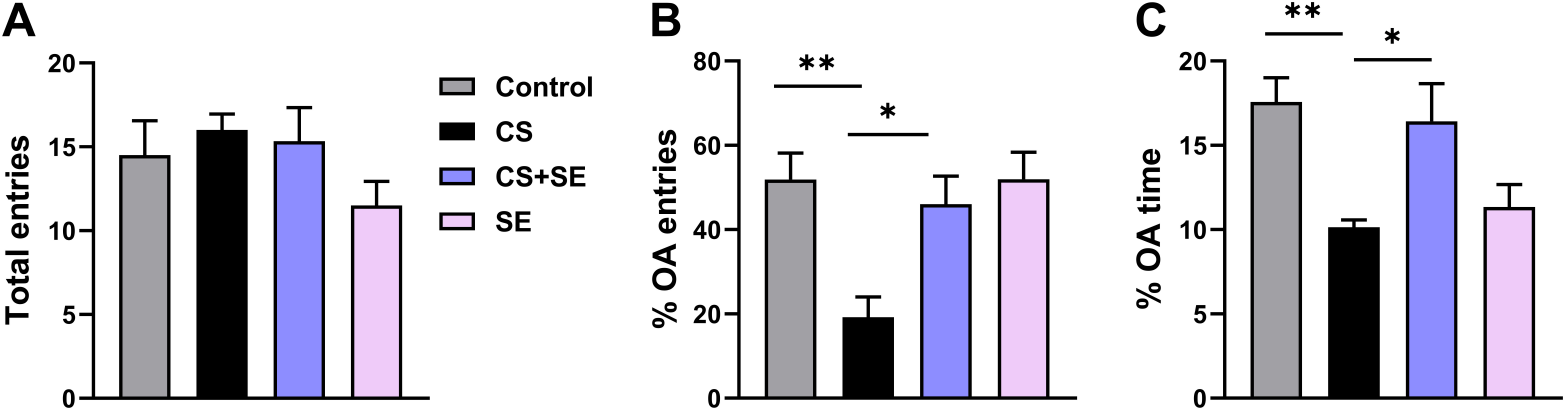
Effect of SE extract on anxiety-like behavior in the elevated plus maze test **(A)** Total arm entries, **(B)** Percentage of OA entries, **(C)** Time spent in OAs. Data are expressed as mean ± SEM. Statistical analysis was performed using one-way ANOVA followed by Bonferroni’s *post-hoc* test. *p* < 0.05 was considered statistically significant. *p < 0.05, **p < 0.01.

Cold stress significantly induced anxiety-like behavior, as observed from EPM and OF tests. In EPM, total arm entries did not differ significantly among the experimental groups [*F*(3, 24) = 0.7677, *p* = 0.5234; Figure 5A], indicating that locomotor activity was not significantly affected. On the other hand, the percentage of OA entries [*F*(3, 24) = 5.757, *p* = 0.0041] was significantly altered across groups. *Post hoc* analysis using Bonferroni’s test showed that percentage OA entries were reduced in CS rats, compared to controls (*p* = 0.0042); however, SE supplementation rescued this phenotype (*p* = 0.0178 vs. CS group; Figure 5B). No significant differences were observed between the control and SE groups, suggesting that SE did not alter baseline anxiety levels. Similarly, the CS+SE group did not differ significantly from the controls, indicating effective reversal of stress-induced anxiety. A similar trend was observed for time spent in OAs [*F*(3, 20) = 5.366, *p* = 0.0071]. The CS group showed marked reduction in OA time compared to the control group (*p* = 0.0090); however, SE supplementation abolished this increase in anxiety-like behavior (*p* = 0.0308 vs. CS group; Figure 5C).

**FIGURE 6:**
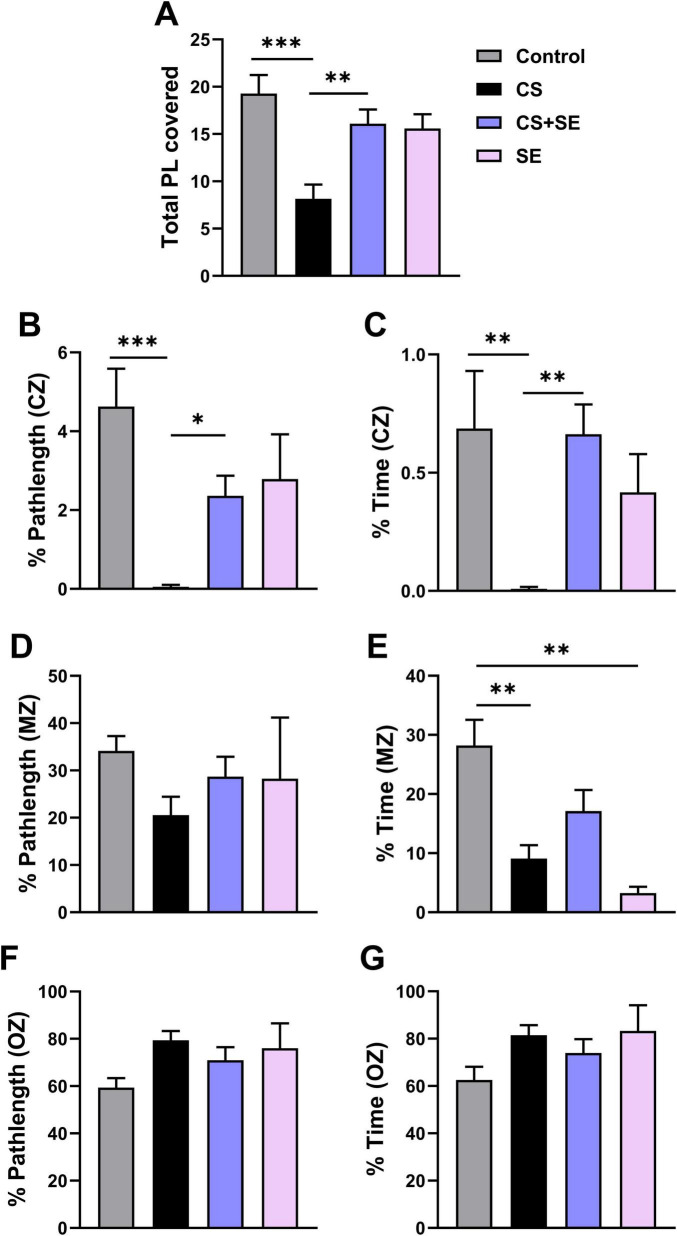
Effect of SE extract on exploratory and anxiety-like behavior in the open field test **(A)** Total pathlength, **(B)** central zone path %, **(C)** central zone time %, **(D)** middle zone path %, **(E)** middle zone time %, **(F)** outer zone path %, **(G)** outer zone time %. Data are expressed as mean ± SEM. Statistical analysis was performed using one-way ANOVA followed by Bonferroni’s *post-hoc* test. *p* < 0.05 was considered statistically significant. *p < 0.05, **p < 0.01, ***p < 0.001.

In OF test, total pathlength was significantly altered across the experimental groups [*F*(3, 25) = 8.238, *p* = 0.0006; Figure 6A]. CS group had significantly reduced total pathlength compared to the control group (*p* = 0.0003). SE treatment improved exploratory activity, as evidenced by a significant increase in total pathlength in the CS+SE group compared to the CS group (*p* = 0.0066). No significant differences were observed between the control and SE groups. Percentage pathlength [*F*(3, 22) = 9.378, *p* = 0.0003] and time spent [*F*(3, 22) = 6.213, *p* = 0.0032] in the CZ also varied significantly across groups. The CS group showed significantly reduced percentage pathlength (*p* = 0.0001) and time spent in CZ (*p* = 0.0095), compared to the control rats, indicating increased anxiety-like behavior. The CS+SE group showed significantly enhanced pathlength covered in the CZ compared to the stress (CS) group (*p* = 0.0369), indicating SE-mediated anxiolysis. Further, SE treatment significantly restored time spent in the CZ (*p* = 0.0032; Figures 6B,C). No significant differences were observed in pathlength covered in the MZ across the animal groups [*F*(3, 23) = 1.695, *p* = 0.1959]. However, time spent in the MZ was significantly affected by animal groups [*F*(3, 21) = 7.610, *p* = 0.0013; Figures 6D,E]. Thus, CS animals had significantly diminished MZ exploration time compared to the controls (*p* = 0.0029). A significant difference was also observed between the control and SE groups (*p* = 0.0027), whereas no difference was found between the CS and CS+SE groups (*p* = 0.4010). No significant differences were observed in pathlength covered (F (3, 25) = 2.765, *p* = 0.0629) or time spent [*F*(3, 25) = 2.335, *p* = 0.0982] in the OZ amongst the experimental groups (Figures 6F,G). Overall, the results suggest that chronic cold temperature exposure induced anxiety-like behavior, and SE treatment partially alleviated these effects.

#### 3.3.3 Oral SE intervention improves spatial working and object recognition memory

**FIGURE 7:**
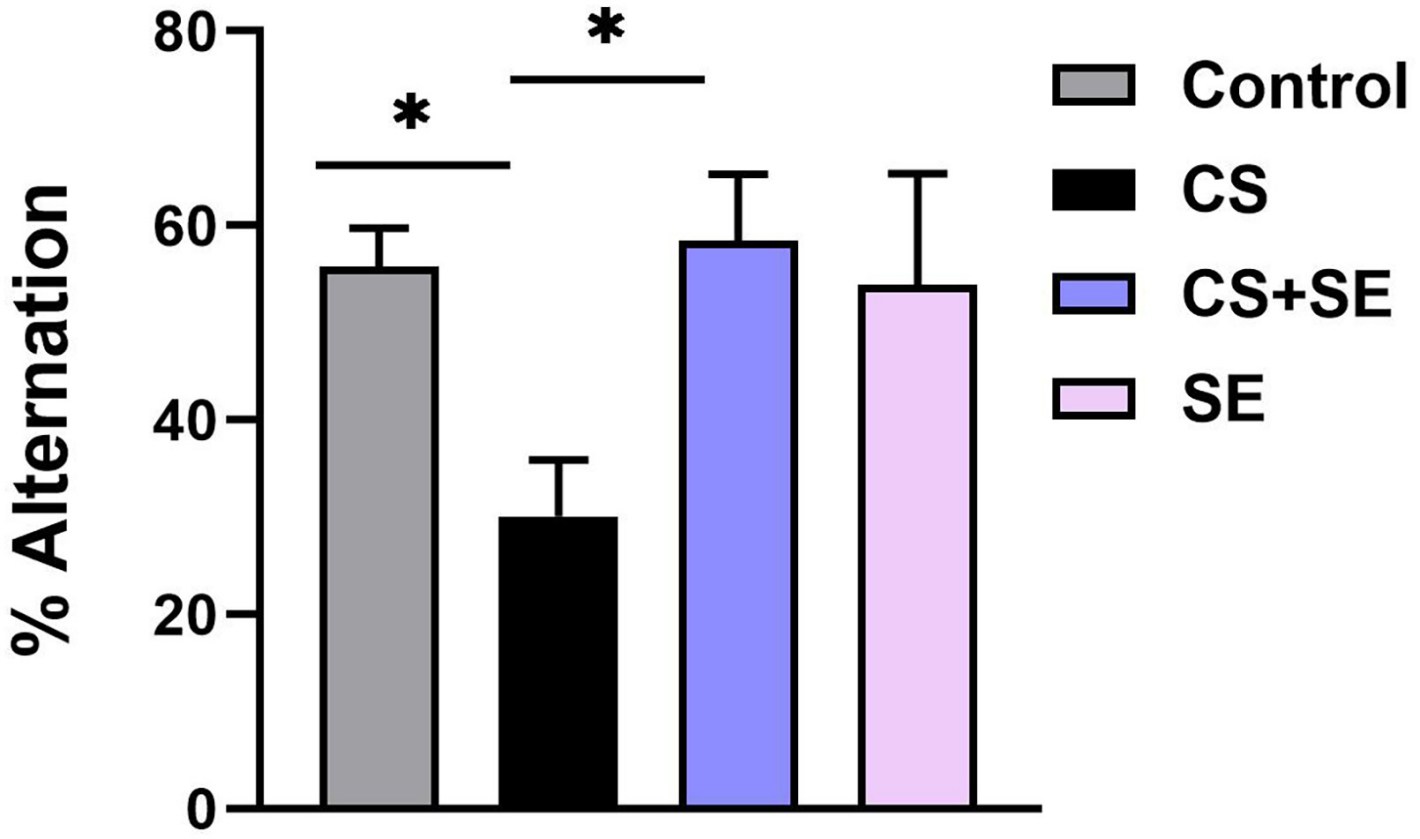
Effect of SE extract on spontaneous alternation behavior in the Y-maze test. Data are expressed as mean ± SEM. Statistical analysis was performed using one-way ANOVA followed by Bonferroni’s *post-hoc* test. *p* < 0.05 was considered statistically significant. *p < 0.05.

Cold stress significantly impaired spatial working memory, as evidenced by a marked reduction in the percentage correct alternation compared with the control group (Figure 7). One-way ANOVA revealed significant effects of animal groups (insult and treatment) on spontaneous correct alternation [*F*(3, 25) = 4.87, *p* = 0.0084]. *Post hoc* analysis using Bonferroni’s multiple comparison test demonstrated that the CS group showed a decrease in percentage correct alternation compared to the controls (*p* = 0.0289). The CS+SE group had higher levels of percentage correct spontaneous alternation compared to the CS group (*p* = 0.0100), indicating restoration of spatial working memory. No significant difference was observed between the control and SE group, suggesting that SE administration did not alter baseline cognitive performance. Similarly, the CS+SE group did not differ significantly from control, indicating near recovery of memory deficits induced by chronic cold temperature exposure.

**FIGURE 8:**
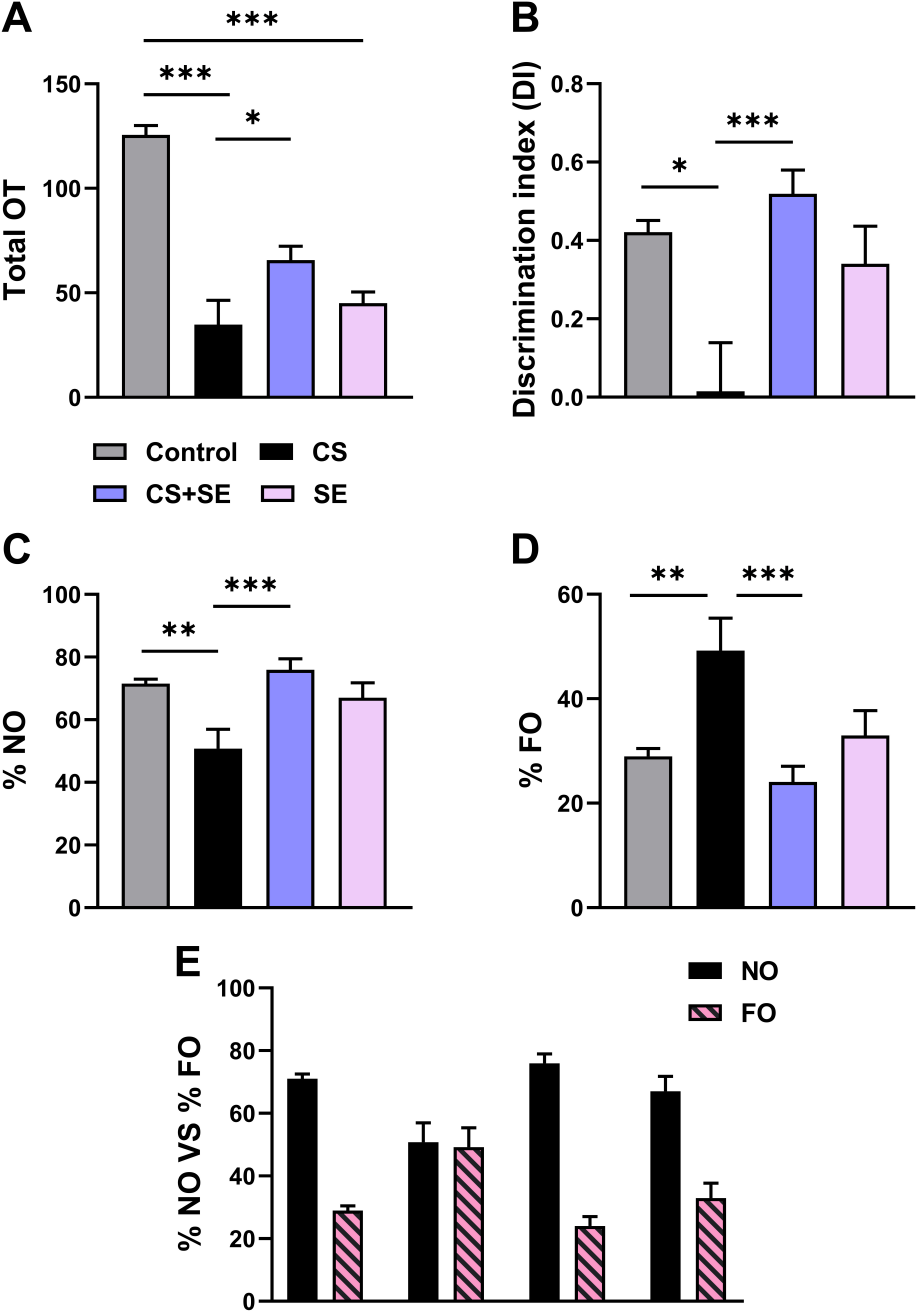
Effect of SE extract on recognition memory in the novel object recognition test. **(A)** Total object exploration time, **(B)** discrimination index, **(C)** percentage of novel object exploration (NO%), **(D)** percentage of familiar object exploration (FO%), **(E)** comparison of NO vs. FO preference. Data are expressed as mean ± SEM. Statistical analysis was performed using one-way ANOVA followed by Bonferroni’s multiple comparisons test for **(A–D)**, and mixed-effects model (REML) for **(E)**. *p < 0.05, **p < 0.01, ***p < 0.001.

The effect of SE treatment on object recognition memory was evaluated using NOR test. The parameters measured included total object exploration time (OT) and percentage time exploring novel object (%NO) and familiar objects (%FO). Discrimination index (DI) calculated as time exploring NO minus time exploring FO divided by OT was also determined. Further, object preference comparison (NO vs. FO) across groups was also undertaken using a mixed effects model with Geisser-Greenhouse correction. Analysis of OT revealed a significant effect of treatment [*F*(3, 25) = 25.60, *p* < 0.0001; Figure 8A). The CS group showed a marked reduction in exploration time compared to the control group (*p* < 0.0001), indicating reduced exploratory drive under chronic cold stress. In contrast, the CS+SE group exhibited a significant increase in OT compared to the stress group (*p* = 0.0467), suggesting partial restoration of exploratory behavior. Significant difference was also observed for the CS+SE (*p* < 0.0001) and SE groups (*p* < 0.0001), compared to the controls.

The percentage of time spent exploring the novel object differed significantly among groups [*F*(3, 25) = 7.589, *p* = 0.0009; Figure 8C]. Cold stress significantly impaired recognition memory, as evidenced by a reduction in %NO (*p* = 0.0036) and a corresponding increase in FO exploration, compared to the controls. SE treatment significantly increased %NO compared to the CS group (*p* = 0.0006), indicating restoration of recognition memory. However, no significant differences in %NO were observed between the control and SE groups. Consistent with the above findings for NO, %FO also showed a significant effect of insult/treatment [*F*(3, 25) = 7.681, *p* = 0.0008; Figure 8D]. The CS group exhibited significantly higher %FO compared to the controls (*p* = 0.0057), reflecting a change of preference from novelty. This increase was significantly reduced in the CS+SE group (*p* = 0.0004), indicating again that SE normalizes object recognition memory in stressed rats.

The DI further confirmed these findings, showing significant changes across animal groups [*F*(3, 25) = 7.681, *p* = 0.0008; Figure 8B]. Pair-wise comparisons indicated that CS animals exhibited repressed DI compared to the control group (*p* = 0.0086). Importantly, the CS+SE group showed significantly improved DI (*p* = 0.0007 vs. CS group). No significant differences were observed between the control and SE treatment groups. To further assess object preference, a mixed-effects analysis was conducted, which revealed significant main effect of object type (NO vs. FO) [*F*(1, 50) = 107.2, *p* < 0.0001; Figure 8E] on object exploration time. While the effect of animal group × object interaction was significant [*F*(3, 50) = 15.36, *p* < 0.0001], the influence of animal group remined non-significant. These results indicate that although animals generally preferred the novel object, this preference was significantly altered by treatment (CS or SE or both) conditions. Specifically, CS induced a diminishment in the ability to discriminate between novel and familiar objects, whereas SE supplementation restored this preference. Collectively, the results from Y-maze and NOR tests conclude that while chronic cold temperature exposure impaired exploratory and memory functions, treatment with SE effectively ameliorated these deficits.

## 4 Discussion

The present study sequentially evaluates the neuroprotective functions of SE in regulating cold stress-induced neuropathological outcomes in aged rats. The initial objective of the study was to evaluate the phytochemical profile of SE in order to ascertain the presence of the key known bioactive constituents as well as to confirm its *ex vivo* antioxidant activity. The presence of key bioactive constituents, crocin and safranal, was confirmed by UV-visible spectroscopy, HPLC, and GC-MS. These findings are consistent with previous reports identifying crocin as the major carotenoid and safranal as the principal volatile compound responsible for saffron’s pharmacological properties (Melnyk et al., 2010; Tarantilis et al., 1994). The presence of aldehydes and ketones reported here was also similar to the previous findings (Carmona et al., 2007). The low DPPH IC_50_ value corresponding to the strong antioxidant activity observed in the present study may be attributed to the presence of bioactive constituents such as crocin and safranal, which have been previously reported to exhibit potent radical scavenging activity through hydrogen-donating mechanisms (Assimopoulou et al., 2005). The total phenolic (75.13 mg GAE/g) and flavonoid content (41.94 mg QE/g) of SE observed in the present study fall within the upper range of values reported previously (Zaazaa et al., 2021). Flavonoids are well-known for their potency to act as free radical scavengers with strong antioxidative activity (Hassanpour and Doroudi, 2023; Kazazić, 2004). Considering this, the higher flavonoid value in the present study can likely be a key contributor to the observed antioxidant potential of the extract. The FTIR analysis also showed the presence of several functional groups corresponding to hydroxyl, carbonyl, aromatic, and glycosidic functional groups, supporting the presence of phenolics, flavonoids, and carotenoid derivatives, which are consistent with previous results (Mykhailenko et al., 2022).

The therapeutic actions of SE supplementation in aged rats challenged with chronic exposure to cold temperature stress were immediately apparent as improvements in body weight of stressed rats across the time frame of the experimental paradigm. Behavioral assessments carried out in the different experimental animal groups revealed that chronic cold stress significantly impaired spatial working and object recognition memory, and induced anxiety-like behavior. These findings are in line with previous reports demonstrating that cold stress exposure disrupts cognitive and emotional regulation. For example, it has been reported that repeated cold stress induces oxidative stress, neuroinflammation, and synaptic dysfunction in the hippocampus, ultimately contributing to anxiety-like behavior and cognitive decline (Qu et al., 2017). Moreover, similar outcomes were observed in other experimental models of chronic cold exposure (Değirmenci et al., 2024), further supporting the validity of the present findings. Importantly, we observed that oral supplementation of SE during the timeframe of the insult effectively ameliorated the neuropathological deficits induced by cold temperature exposure. This was observed as restoration of Y-maze performance, recovery of NOR task deficits, and anxiolytic effects observed in the EPM and OF tests. This is in agreement with previous studies reporting that crocin and safranal improve learning and memory in rodents subjected to chronic cerebral hypoperfusion (Hosseinzadeh et al., 2012). Interestingly, SE supplementation also improved cold stress-induced repression of total pathlength covered in the OF arena. However, total arm entries in EPM and Y-maze tests were not affected by either cold stress or by SE treatment. This indicates that general locomotion and exploratory activities are largely intact upon induction of chronic cold stress. Further, this also suggests that SE seems to exert a specific effect on emotional and memory pathways, rather than stimulating nonspecific motor pathways.

Several preclinical studies have established the anxiolytic and neuroprotective effects of saffron and its bioactive carotenoids, such as crocin, primarily under conventional psychological stress and mood-disorder paradigms, including restraint stress (Ghadrdoost et al., 2011) and chronic unpredictable mild stress (Zhou et al., 2025). In the human clinical perspective, saffron supplementation has been implicated in broad improvements in psychological wellbeing (Lopresti et al., 2025). Studies also suggest that it exhibits therapeutic efficacy comparable to conventional selective serotonin reuptake inhibitors (SSRIs) (Shafiee et al., 2025), and mitigates emotional stress during acute psychosocial challenges (Jackson et al., 2021) and neurobehavioral disruptions (Blasco-Fontecilla et al., 2022). In the present study, we evaluated the anxiolytic effect of SE in an aged rodent model exposed to chronic cold stress. Exposure to extreme temperatures represents an environmentally relevant neurophysiological stress associated with mood and cognitive impairment in susceptible populations. Although the precise underlying cellular and molecular mechanisms were not elucidated, our findings highlight the potential translational and future clinical relevance of saffron for aged populations exposed to chronic but sublethal cold conditions. Given that cold stress is known to induce oxidative imbalance and reactive oxygen species (ROS) generation in the brain, the observed antioxidant potential of the extract likely plays a central role in the neuroprotective effects observed in this study (Melnyk et al., 2010). Mechanistically, the observed behavioral improvements may be attributed to the multi-target actions of saffron constituents, including antioxidant activity, modulation of neurotransmitter systems and neuroinflammatory pathways associated with cold stress-induced brain injury. Future studies should focus on elucidating the molecular mechanisms underlying the observed behavioral effects of SE, for e.g., its influence on stress-responsive pathways (Abdian et al., 2024; Anaeigoudari et al., 2023), and synaptic integrity and neuronal survival pathways (Xu et al., 2019), especially in the context of aging (Zhang et al., 2025).

Clinically, our findings are of particular interest for vulnerable populations chronically exposed to cold environments, including elderly populations, military personnel, outdoor workers and migrant populations. The study supports the potential application of saffron as an adjunct nutritional strategy for stress-associated cognitive and memory dysfunction. Furthermore, the distinct behavioral profile observed in the present study suggests that SE may hold a significant promise as a candidate for multidrug or functional combination therapies aimed at improving behavioral resilience, while potentially reducing the associated side effects caused by high-dose conventional anxiolytic or cognitive-enhancing medications.

## 5 Conclusion

The present work provides comprehensive evidence of the potential effect of SE in addressing behavioral deficits such as anxiety and cognitive impairments associated with chronic cold stress exposure. While the neuroprotective effects of saffron have been known in other pathological models, the current findings extend its relevance to cold stress paradigms, an area that remains relatively underexplored. The observed behavioral improvements, together with its rich phytochemical composition and antioxidant capacity, suggest that saffron may effectively counteract environmental stress-induced neurobiological alterations. Importantly, these results highlight the potential role of saffron as a multi-target intervention in environmental stress conditions such as cold exposure. This opens new avenues for future research exploring its application in populations exposed to extreme climates, thereby broadening its therapeutic scope in stress-related neuropsychiatric disorders.

## Data Availability

The original contributions presented in this study are included in the article/supplementary material, further inquiries can be directed to the corresponding author.

## References

[B1] Abbaszade-Cheragheali A. Beheshti F. Kakhki S. Khatibi S. R. Dehnokhalaji F. Akbari E. et al. (2022). Crocin, the main active saffron (*Crocus sativus* L.) constituent, as a potential candidate to prevent anxiety and depressive-like behaviors induced by unpredictable chronic mild stress. *Neurosci. Lett.* 791:136912. 10.1016/j.neulet.2022.136912 36244518

[B2] Abdian S. Fakhri S. Moradi S. Z. Khirehgesh M. R. Echeverría J. (2024). Saffron and its major constituents against neurodegenerative diseases: A mechanistic review. *Phytomedicine* 135:156097. 10.1016/j.phymed.2024.156097 39577115

[B3] Ahmad F. Mein H. Jing Y. Zhang H. Liu P. (2021). Behavioural functions and cerebral blood flow in a P301S tauopathy mouse model: A time-course study. *Int. J. Mol. Sci.* 22:9727. 10.3390/ijms22189727 34575885 PMC8468775

[B4] Alam P. Elkholy S. F. Hosokawa M. Mahfouz S. A. Sharaf-Eldin M. A. (2016). Simultaneous extraction and rapid HPLC based quantification of crocin and safranal in saffron (*Crocus sativus* L.). *Int. J. Pharm. Pharm. Sci.* 8 224–227. 10.22159/ijpps.2016v8i10.12172

[B5] Albernaz-Mariano K. A. Malta M. B. Bueno-De-Camargo L. M. Duque E. D. A. Novaes L. S. Dragunas G. et al. (2025). Unpredictable stress boosts perceptual learning and alters glucocorticoid and norepinephrine receptors in rats’ dorsal hippocampus. *Transl. Psychiatry* 15:508. 10.1038/s41398-025-03716-6 41258066 PMC12660680

[B6] Alnaddaf L. M. Ksaier M. N. Bamsaoud S. F. (2023). Evaluation of *Crocus sativus* L. quality using FTIR spectroscopy *Damascus Univ. J. Nat. Appl. Sci.* 20 9–13.

[B7] Anaeigoudari F. Anaeigoudari A. Kheirkhah-Vakilabad A. (2023). A review of therapeutic impacts of saffron (*Crocus sativus* L.) and its constituents. *Physiol. Rep.* 11 e15785. 10.14814/phy2.15785 37537722 PMC10400758

[B8] Asgary S. (2021). Saffron and depression. *Bioact. Compd. Health Dis.* 4 90–97. 10.31989/bchd.v4i5.808

[B9] Assimopoulou A. N. Sinakos Z. Papageorgiou V. P. (2005). Radical scavenging activity of *Crocus sativus* L. extract and its bioactive constituents. *Phytother. Res.* 19 997–1000. 10.1002/ptr.1749 16317646

[B10] Baliyan S. Mukherjee R. Priyadarshini A. Vibhuti A. Gupta A. Pandey R. P. et al. (2022). Determination of antioxidants by DPPH radical scavenging activity and quantitative phytochemical analysis of *Ficus religiosa*. *Molecules* 27:1326. 10.3390/molecules27041326 35209118 PMC8878429

[B11] Ben-Azu B. Chidebe E. O. Toloyai P. Y. Annafi O. S. Oritsemuelebi B. Asiwe J. et al. (2024). Adaptogenic action of diosgenin againsts chronic unpredictable mild stress-induced neuroimmune dysfunction of HPA axis reverses psychiatric behavior in mice. *Clin. Tradit. Med. Pharmacol.* 5:200148. 10.1016/j.ctmp.2024.200148

[B12] Blasco-Fontecilla H. Moyano-Ramírez E. Méndez-González O. Rodrigo-Yanguas M. Martin-Moratinos M. Bella-Fernández M. (2022). Effectivity of saffron extract (Saffr’Activ^®^) on treatment for children and adolescents with Attention Deficit/Hyperactivity Disorder (ADHD): A clinical effectivity study. *Nutrients* 14:4046. 10.3390/nu14194046 36235697 PMC9573091

[B13] Bowman R. E. Beck K. D. Luine V. N. (2003). Chronic stress effects on memory: Sex differences in performance and monoaminergic activity. *Horm. Behav.* 43 48–59. 10.1016/S0018-506X(02)00022-3 12614634

[B14] Campos A. C. Fogaça M. V. Aguiar D. C. Guimarães F. S. (2013). Animal models of anxiety disorders and stress. *Rev. Bras. Psiquiatr.* 35 (Suppl. 2), S101–S111. 10.1590/1516-4446-2013-1139 24271222

[B15] Carmona M. Zalacain A. Salinas M. R. Alonso G. L. (2007). A new approach to saffron aroma. *Crit. Rev. Food Sci. Nutr.* 47 145–159. 10.1080/10408390600626511 17364699

[B16] Cerdá-Bernad D. Costa L. Serra A. T. Bronze M. R. Valero-Cases E. Pérez-Llamas F. et al. (2022). Saffron against neuro-cognitive disorders: An overview of its main bioactive compounds, their metabolic fate and potential mechanisms of neurological protection. *Nutrients* 14:5368. 10.3390/nu14245368 36558528 PMC9781906

[B17] Chauhan S. Tiwari A. Verma A. Padhan P. K. Verma S. Gupta P. C. (2024). Exploring the potential of saffron as a therapeutic agent in depression treatment: A comparative review. *Yale J. Biol. Med.* 97 365–381. 10.59249/XURF4540 39351321 PMC11426294

[B18] Coates J. (2006). “Interpretation of Infrared Spectra, a Practical Approach,” in *Encyclopedia of Analytical Chemistry*, ed. Meyers R. A. (Chichester: John Wiley & Sons Ltd), 10.1002/9780470027318.a5606

[B19] Değirmenci M. D. Çalışkan H. Güneş E. (2024). Effects of chronic intermittent cold stress on anxiety-depression-like behaviors in adolescent rats. *Behav. Brain Res.* 472:115130. 10.1016/j.bbr.2024.115130 38936426

[B20] El Marzouki H. Aboussaleh Y. Najimi M. Chigr F. Ahami A. (2021). Effect of cold stress on neurobehavioral and physiological parameters in rats. *Front. Physiol.* 12:660124. 10.3389/fphys.2021.660124 34603068 PMC8485037

[B21] El Midaoui A. Ghzaiel I. Vervandier-Fasseur D. Ksila M. Zarrouk A. Nury T. et al. (2022). Saffron (*Crocus sativus* L.): A source of nutrients for health and for the treatment of neuropsychiatric and age-related diseases. *Nutrients* 14:597. 10.3390/nu14030597 35276955 PMC8839854

[B22] Falla M. Masè M. Dal Cappello T. Micarelli A. van Veelen M. J. Roveri G. et al. (2026). Cold stress impacts cognitive performance in healthy volunteers: Results from a randomized, controlled, cross-over study. *Sci. Rep.* 16:7013. 10.1038/s41598-026-38048-y 41634322 PMC12921262

[B23] Foschi M. Tozzi L. Di Donato F. Biancolillo A. D’Archivio A. A. (2022). A novel FTIR-based chemometric solution for the assessment of saffron adulteration with non-fresh stigmas. *Molecules* 28:0033. 10.3390/molecules28010033 36615229 PMC9821794

[B24] Frusciante L. Geminiani M. Shabab B. Olmastroni T. Scavello G. Rossi M. et al. (2024). Exploring the antioxidant and anti-inflammatory potential of saffron (*Crocus sativus*) tepals extract within the circular bioeconomy. *Antioxidants* 13:1082. 10.3390/antiox13091082 39334741 PMC11428576

[B25] Ghadrdoost B. Vafaei A. A. Rashidy-Pour A. Hajisoltani R. Bandegi A. R. Motamedi F. et al. (2011). Protective effects of saffron extract and its active constituent crocin against oxidative stress and spatial learning and memory deficits induced by chronic stress in rats. *Eur. J. Pharmacol.* 667 222–229. 10.1016/j.ejphar.2011.05.012 21616066

[B26] Hale G. Good M. (2005). Impaired visuospatial recognition memory but normal object novelty detection and relative familiarity judgments in adult mice expressing the APPswe Alzheimer’s disease mutation. *Behav. Neurosci.* 119 884–891. 10.1037/0735-7044.119.4.884 16187817

[B27] Hassanpour S. H. Doroudi A. (2023). Review of the antioxidant potential of flavonoids as a subgroup of polyphenols and partial substitute for synthetic antioxidants. *Avicenna J. Phytomed.* 13 354–376. 10.22038/AJP.2023.21774 37663389 PMC10474916

[B28] Hoffman A. N. Krigbaum A. Ortiz J. B. Mika A. Hutchinson K. M. Bimonte-Nelson H. A. et al. (2011). Recovery after chronic stress within spatial reference and working memory domains: Correspondence with hippocampal morphology. *Eur. J. Neurosci.* 34 1023–1030. 10.1111/j.1460-9568.2011.07820.x 21884554

[B29] Hosseinzadeh H. Sadeghnia H. R. Ghaeni F. A. Motamedshariaty V. S. Mohajeri S. A. (2012). Effects of saffron (*Crocus sativus* L.) and its active constituent, crocin, on recognition and spatial memory after chronic cerebral hypoperfusion in rats. *Phytother. Res.* 26 381–386. 10.1002/ptr.3566 21774008

[B30] Hu Y. Liu Y. Li S. (2022). Effect of acute cold stress on neuroethology in mice and establishment of its model. *Animals* 12:2671. 10.3390/ani12192671 36230412 PMC9559653

[B31] Jackson P. A. Forster J. Khan J. Pouchieu C. Dubreuil S. Gaudout D. et al. (2021). Effects of saffron extract supplementation on mood, well-being, and response to a psychosocial stressor in healthy adults: A randomized, double-blind, parallel group, clinical trial. *Front. Nutr.* 7:606124. 10.3389/fnut.2020.606124 33598475 PMC7882499

[B32] Jahromi G. P. Jangravi Z. Hadipour M. Shirvani H. Afarinesh M. R. Meftahi G. H. (2023). Comparative effects of the alcoholic extract of *Terminalia chebula* and crocin on stress induced anxiety like behavior and memory impairment in male rats. *Acta Neurobiol. Exp.* 83 179–193. 10.55782/ane-2023-016 37493534

[B33] Jänicke B. Coper H. (1996). “Tests in Rodents for Assessing Sensorimotor Performance During Aging,” in *Aging and Cognitive Processes*, eds Hof P. R. Mobbs C. V. (Amsterdam: Elsevier), 201–233. 10.1016/S0166-4115(96)80010-0

[B34] Jayakumar S. Raghunath G. Ilango S. Vijayakumar J. Vijayaraghavan R. (2017). Effect of fluoxetine on the hippocampus of *Wistar albino* rats in cold restraint stress model. *J. Clin. Diagn. Res.* 11 AF01–AF06. 10.7860/JCDR/2017/26958.9953 28764145 PMC5535338

[B35] José Bagur M. Alonso Salinas G. Jiménez-Monreal A. Chaouqi S. Llorens S. Martínez-Tomé M. et al. (2017). Saffron: An old medicinal plant and a potential novel functional food. *Molecules* 23:30. 10.3390/molecules23010030 29295497 PMC5943931

[B36] Kazazić S. P. (2004). Antioxidative and antiradical activity of flavonoids. *Arh. Hig. Rada Toksikol.* 55 279–290. 15584555

[B37] Khattak M. Malik M. O. Usman R. Habib S. H. Saddique U. et al. (2023). Developing chronic unpredictable/alternating stress model in *Wistar albino* rats. *J. Popul. Ther. Clin. Pharmacol.* 30 223–232. 10.53555/jptcp.v30i19.3623

[B38] Khodaie L. Bamdad S. Delazar A. Nazemiyeh H. (2012). Antioxidant, total phenol and flavonoid contents of two *Pedicularis L*. species from eastern Azerbaijan. *Iran. Bioimpacts* 2 43–57. 10.5681/bi.2012.006 23678441 PMC3648916

[B39] Khorasany A. R. Hosseinzadeh H. (2016). Therapeutic effects of saffron (*Crocus sativus* L.) in digestive disorders: A review. *Iran. J. Basic Med. Sci.* 19 455–469. 27403251 PMC4923465

[B40] Kim C. Y. Ko K. Choi S. H. Jo M. Kim J. Yoon S. et al. (2023). Effects of saffron extract (Affron^®^) with 100 mg/kg and 200 mg/kg on hypothalamic–pituitary–adrenal axis and stress resilience in chronic mild stress-induced depression in Wistar rats. *Nutrients* 15:4855. 10.3390/nu15234855 38068714 PMC10707924

[B41] Kline S. A. Mega M. S. (2020). Stress-induced neurodegeneration: The potential for coping as neuroprotective therapy. *Am. J. Alzheimers Dis. Other Demen.* 35:1533317520960873. 10.1177/1533317520960873 32969239 PMC10623922

[B42] Lage M. Cantrell C. L. (2009). Quantification of saffron (*Crocus sativus* L.) metabolites crocins, picrocrocin and safranal for quality determination of the spice grown under different environmental Moroccan conditions. *Sci. Hortic.* 121 366–373. 10.1016/j.scienta.2009.02.017

[B43] Lei A. A. Phang V. W. X. Lee Y. Z. Kow A. S. F. Tham C. L. Ho Y. C. et al. (2025). Chronic stress-associated depressive disorders: The impact of HPA axis dysregulation and neuroinflammation on the hippocampus—a mini review. *Int. J. Mol. Sci.* 26:2940. 10.3390/ijms26072940 40243556 PMC11988747

[B44] Li L. Wang K. (2025). Integrative effects of saffron and physical activity on endurance performance, quality of life, cognitive, emotional, and metabolic outcomes in age-related and neurodegenerative diseases. *Front. Nutr.* 12:1698135. 10.3389/fnut.2025.1698135 41393939 PMC12695556

[B45] Lopresti A. L. Smith S. J. Marx W. Díez-Municio M. Morán-Valero M. I. (2025). An examination into the effects of a saffron extract (Affron^®^) on mood and general wellbeing in adults experiencing low mood: A randomized, double-blind, placebo-controlled trial. *J. Nutr.* 155 2300–2311. 10.1016/j.tjnut.2025.05.024 40414301

[B46] Martínez Lozada P. S. Leon-Rojas J. E. (2025). Neurological and mental health in the era of climate change: Mechanisms, clinical impacts, and adaptation. *Front. Public Health* 13:1630975. 10.3389/fpubh.2025.1630975 41018788 PMC12460379

[B47] McEwen B. S. (2006). Protective and damaging effects of stress mediators: Central role of the brain. *Dial. Clin. Neurosci.* 8 367–381. 10.31887/DCNS.2006.8.4/bmcewen 17290796 PMC3181832

[B48] McEwen B. S. Akil H. (2020). Revisiting the stress concept: Implications for affective disorders. *J. Neurosci.* 40 12–21. 10.1523/JNEUROSCI.0733-19.2019 31896560 PMC6939488

[B49] Melnyk J. P. Wang S. Marcone M. F. (2010). Chemical and biological properties of the world’s most expensive spice: Saffron. *Food Res. Int.* 43 1981–1989. 10.1016/j.foodres.2010.07.033

[B50] Mir T. U. G. Singh J. Shukla S. (2022). Quantification of apocarotenoids in commercial Indian (Kashmiri) saffron using UV-Vis spectroscopy and HPLC analysis. *Acta Agric. Slov.* 118 1–11. 10.14720/aas.2022.118.3.2333

[B51] Mirhadi E. Nassirli H. Malaekeh-Nikouei B. (2020). An updated review on therapeutic effects of nanoparticle-based formulations of saffron components (safranal, crocin, and crocetin). *J. Pharm. Investig.* 50 47–58. 10.1007/s40005-019-00435-1

[B52] Mishra R. Jain V. (2025). Exploring the potential of traditional herbal medicine in the management of central nervous system disorders. *Phytomedicine Plus* 5:100896. 10.1016/j.phyplu.2025.100896

[B53] Muller M. D. Gunstad J. Alosco M. L. Miller L. A. Updegraff J. Spitznagel M. B. et al. (2012). Acute cold exposure and cognitive function: Evidence for sustained impairment. *Ergonomics* 55 792–798. 10.1080/00140139.2012.665497 22506538 PMC3375336

[B54] Mykhailenko O. Bezruk I. Volochai V. Mishchenko V. Ivanauskas L. Georgiyants V. (2022). Phytochemical analysis and antioxidant activity of *Crocus speciosus* leaves. *Phyton.* 91 207–221. 10.32604/phyton.2022.016458

[B55] Naim N. Ennahli N. Hanine H. Lahlali R. Tahiri A. Fauconnier M. L. et al. (2022a). ATR-FTIR spectroscopy combined with DNA barcoding and GC-MS to assess the quality and purity of saffron (*Crocus sativus* L.). *Vib. Spectrosc.* 123:103446. 10.1016/j.vibspec.2022.103446

[B56] Naim N. Fauconnier M. L. Ennahli N. Tahiri A. Baala M. Madani I. et al. (2022b). Chemical composition profiling and antifungal activity of saffron petal extract. *Molecules* 27:8742. 10.3390/molecules27248742 36557875 PMC9787665

[B57] Navarro J. A. Gavito A. Rivas S. Rodríguez-Martín A. Baixeras E. Decara J. et al. (2026). Safranal-standardized saffron extract improves metabolic, cognitive, and anxiolytic outcomes in aged mice via hypothalamic–amygdalar peptide modulation. *Nutrients* 18:291. 10.3390/nu18020291 41599903 PMC12845050

[B58] Pellow S. Chopin P. File S. E. Briley M. (1985). Validation of open:closed arm entries in an elevated plus-maze as a measure of anxiety in the rat. *J. Neurosci. Methods* 14 149–167. 10.1016/0165-0270(85)90031-7 2864480

[B59] Priya V. Ahmad F. (2026). A narrative review on rodent models of neuropsychopathology associated with low temperature exposure. *Neuropsychiatr. Dis. Treat.* 22 1–19. 10.2147/NDT.S571418 41908336 PMC13019168

[B60] Qu T. T. Deng J. X. Li R. L. Cui Z. J. Wang X. Q. Wang L. et al. (2017). Stress injuries and autophagy in mouse hippocampus after chronic cold exposure. *Neural Regen. Res.* 12 440–446. 10.4103/1673-5374.202932 28469659 PMC5399722

[B61] Rezaee R. Hosseinzadeh H. (2013). Safranal: From an aromatic natural product to a rewarding pharmacological agent. *Iran. J. Basic Med. Sci.* 16 12–26. 23638289 PMC3637901

[B62] Ríos J. L. Recio M. C. Giner R. M. Máñez S. (1996). An update review of saffron and its active constituents. *Phytother. Res.* 10 189–193. 10.1002/(SICI)1099-1573(199605)10:3<189::AID-PTR754>3.0.CO;2-C

[B63] Santori A. Morena M. Hill M. N. Campolongo P. (2020). Hippocampal 2-arachidonoyl glycerol signaling regulates time-of-day- and stress-dependent effects on rat short-term memory. *Int. J. Mol. Sci.* 21:7316. 10.3390/ijms21197316 33023013 PMC7582511

[B64] Scholz E. (1984). *Karl Fischer Titration.* Berlin: Springer Berlin Heidelberg, 10.1007/978-3-642-69989-4

[B65] Shafat A. M. Javeed I. A. B. Rouf Ahmad B. Bilal A. B. Hafiz ul I. Shakeel A. D. et al. (2021). “Cytogenetic and bioactive attributes of Crocus sativus (Saffron): A tool to unfold its medicinal mystery,” in *Medicinal and Aromatic Plants*, eds Aftab T. Hakeem K. R. (Amsterdam: Elsevier), 145–167. 10.1016/B978-0-12-819590-1.00007-0

[B66] Shafiee A. Jafarabady K. Seighali N. Mohammadi I. Rajai Firouz, Abadi S. et al. (2025). Effect of saffron versus selective serotonin reuptake inhibitors (SSRIs) in treatment of depression and anxiety: A meta-analysis of randomized controlled trials. *Nutr. Rev.* 83 e751–e761. 10.1093/nutrit/nuae076 38913392

[B67] Siddiqui S. A. Ali Redha A. Snoeck E. R. Singh S. Simal-Gandara J. Ibrahim S. A. et al. (2022). Anti-depressant properties of crocin molecules in saffron. *Molecules* 27:2076. 10.3390/molecules27072076 35408474 PMC9000812

[B68] Smolander J. (2002). Effect of cold exposure on older humans. *Int. J. Sports Med.* 23 86–92. 10.1055/s-2002-20137 11842354

[B69] Stuart B. (2004). *Infrared Spectroscopy: Fundamentals and Applications.* Chichester: John Wiley & Sons Ltd, 10.1002/0470011149

[B70] Suresh M. Chandrasekhar M. Chandrasekhar N. Kondam A. Madhuri B. A. Gajalakshmi G. (2013). A study on behavioural changes induced by cold water stress in Swiss albino mice. *Int. J. Med. Res. Health Sci.* 2 505–510. 10.5958/j.2319-5886.2.3.088

[B71] Tao W. Ruan J. Wu R. Zhao M. Zhao T. Qi M. et al. (2023). A natural carotenoid crocin exerts antidepressant action by promoting adult hippocampal neurogenesis through Wnt/β-catenin signaling. *J. Adv. Res.* 43 219–231. 10.1016/j.jare.2022.02.015 36585110 PMC9811320

[B72] Tarantilis P. A. Morjani H. Polissiou M. Manfait M. (1994). Inhibition of growth and induction of differentiation of promyelocytic leukemia (HL-60) by carotenoids from *Crocus sativus* L. *Anticancer Res.* 14 1913–1918. 7847826

[B73] Valle García-Rodríguez M. Serrano-Díaz J. Tarantilis P. A. López-Córcoles H. Carmona M. Alonso G. L. (2014). Determination of saffron quality by high-performance liquid chromatography. *J. Agric. Food Chem.* 62 8068–8074. 10.1021/jf5019356 25075549

[B74] van der Kolk B. A. (1994). The body keeps the score: Memory and the evolving psychobiology of posttraumatic stress. *Harv. Rev. Psychiatry* 1 253–265. 10.3109/10673229409017088 9384857

[B75] Watkins S. L. Castle P. Mauger A. R. Sculthorpe N. Fitch N. Aldous J. et al. (2014). The effect of different environmental conditions on the decision-making performance of soccer goal line officials. *Res. Sports Med.* 22 425–437. 10.1080/15438627.2014.948624 25295479

[B76] Xu B. Lang L. M. Lian S. Guo J. R. Wang J. F. Yang H. M. et al. (2019). Oxidation stress-mediated MAPK signaling pathway activation induces neuronal loss in the CA1 and CA3 regions of the hippocampus of mice following chronic cold exposure. *Brain Sci.* 9:273. 10.3390/brainsci9100273 31614701 PMC6826747

[B77] Yamaguchi M. (ed.) (2012). *Carotenoids: Properties, Effects and Diseases.* Hauppauge, NY: Nova Science Publishers, Inc.

[B78] Yang X. Chen X. Fu Y. Luo Q. Du L. Qiu H. et al. (2018). Comparative efficacy and safety of *Crocus sativus* L. for treating mild to moderate major depressive disorder in adults: A meta-analysis of randomized controlled trials. *Neuropsychiatr. Dis. Treat.* 14 1297–1305. 10.2147/NDT.S157550 29849461 PMC5967372

[B79] Yaribeygi H. Panahi Y. Sahraei H. Johnston T. P. Sahebkar A. (2017). The impact of stress on body function: A review. *Excli J.* 16 1057–1072. 10.17179/excli2017-480 28900385 PMC5579396

[B80] Zaazaa L. Naceiri Mrabti H. -Dra A. Bendahbia K. Hami H. Soulaymani A. et al. (2021). Determination of mineral composition and phenolic content and investigation of antioxidant, antidiabetic, and antibacterial activities of *Crocus sativus* L. aqueous stigmas extracts. *Adv. Pharmacol. Pharm. Sci.* 2021:7533938. 10.1155/2021/7533938 34195613 PMC8181092

[B81] Zhang Y. Zhang X. Guo J. Dai W. Chen S. Huang L. et al. (2025). Stressors-induced cognitive dysfunction during aging: Mechanisms and future challenges. *Front. Aging Neurosci.* 17:1630982. 10.3389/fnagi.2025.1630982 41200271 PMC12585983

[B82] Zhou J. Hu Y. Jing P. (2025). Antidepressant-like effect of saffron (*Crocus sativus* L.) in mice exposed to chronic unpredictable mild stress via attenuating neuroinflammation and recovering neuroplasticity. *Mol. Nutr. Food Res.* 69:2400702. 10.1002/mnfr.70201 40772362

[B83] Ziani A. Bekkouch O. Ouahhoud S. Baddaoui S. Ben’Mbarek S. Bekkouch A. et al. (2025). Phytochemistry, biological activities, molecular mechanisms, and toxicity of saffron (*Crocus sativus* L.): A comprehensive overview. *Antioxidants* 14:1433. 10.3390/antiox14121433 41462633 PMC12729493

